# Elongated styloid process: An epidemiological study on digital panoramic radiographs

**DOI:** 10.4317/jced.54370

**Published:** 2017-12-01

**Authors:** Giovanni Bruno, Alberto De Stefani, Paolo Balasso, Sergio Mazzoleni, Antonio Gracco

**Affiliations:** 1 Department of Neuroscience, School of Dentistry, University of Padua, Padua, Italy; 2 Department of Management and Engineering, University of Padova

## Abstract

**Background:**

The styloid process is a projecton of the temporal bone, its lenght is between 20 to 30 mm, when it is longer than 30 mm it is defined elongated styloid process. The aim of this study is an epidemiological evaluation of 1003 digital panoramic radiographs in an Italian population between 5 and 90 years old.

**Material and Methods:**

This is a retrospective analysis and the radiographs were selected from the Complex Operating Unit of Dentistry of Padua University Hospital database. The radiographs were performed using a Sirona Ortophos XG and the styloid process length was measured using the measuring tool of Sidexis Software. It was measured from the point where it left the temporal bone plate to its tip. Styloid processes measuring more than 30 mm were considered elongated. Chi-squared test, Fligner-Killeen test, Shapiro-Wilk test and t-test with Welch correction were performed.

**Results:**

In the study 33.40% of the patients showed an elongated styloid process.

**Conclusions:**

The number of patients with elongated styloid process and the mean length of the process increase with the age confirming the chronic development of the calcification described in literature. No statistically significant correlation is found between the presence of elongated styloid process and the gender and affected side (bilateral or unilateral).

** Key words:**Elongated styloid process, panoramic radiograph, epidemiological study, Eagle’s syndrome.

## Introduction

The styloid process is a projection of the temporal bone, it is cylindrical in shape and it is located in front of the stylmastoid foramen and it has a connection with the hyoid bone through the styloid ligament. It is a part of the splancnocranium for its embriological develompment: it derives from the second branchial arch, in the Reichert’s cartilage. The lenght of this process s between 20 to 30 mm and when it is longer than 30 mm it is defined elongated styloid process. This condition is a common aspect in the population and it is usually not associated with any symptoms or other clinical aspects. Some patients that report an elongated styloid process can be affected by orofacial and neck pain during deglution, mouth opening and head rotation. The elongated styloid process associated with these symptoms characterizes the Eagle’s syndrome. Only between 4% and 10% of the people presenting the elongation effectively present Eagle’s syndrome symptoms.

The diagnosis of this condition is not easy for the clinician and it is often misdiagnosed due to its vague symptomatology (1): the aspects of this syndrome are often confused with other orofacial disease like as tooth disease, temporomandibular disorders, impacted third molars (2). There are some studies reported in literature that evaluate the aspects of the syloid process. Different methods have been evaluated: human dry skull (3), digital panoramic radiographs (4,5) computed tomography (CT) (6) and cone beam computed tomography (CBCT) (7). Digital panoramic radiographs show an easier interpretation than other methods and they are the preferable procedure for epidemiological studies and for the first diagnosis.

The prevalence of elongated styloid process has been study in different populations using digital panoramic radiographs and a high variability has been found (4,8). A previous study that evaluates the prevalence of elongated styloid process in an Italian population found a 33% of elongation (5). At the end of that study the authors suggest that other studies could be made with a bigger sample size and to evaluate the increase in lenght of styloid process with advancing age.

The aim of this study is an epidemiological evaluation of the elongated styloid process in the Italian population, to evaluate the difference of elongation between different age groups and if a progressive increase of the elongation with the increasing age can be found.

## Material and Methods

This study was performed as a retrospective analysis on digital panoramic radiographs of 1003 (452 males and 551 females) Italian patients between 5 and 90 years old. It was decided to divide the sample size in four different age groups: 165 patients younger than 18 years old (96 females and 69 males), 199 patients between 18 and 35 years old (118 females and 81 males), 214 patients between 36 and 53 (110 females and 104 males) and finally 425 patients older than 54 years old (227 females and 198 males). Note that the males and females are similarly distributed by the age groups even if the proportion of females in the age groups “≤ 17” and “17-35” are slightly higher than the other groups. We applied a chi-squared test in order to confirm whether the proportion of genders is significantly equal among the age groups. A p-value higher than 0.05 (p-value = 0.29) did not rejected the null hypothesis according to which the proportions are equal among the age groups.

The study was preliminary approved by the local ethical committee. The radiographs were selected from the Complex Operating Unit of Dentistry of Padua University Hospital database.

All the radiographs evaluated were taken during the last five years always by the same operator. Patients were positioned with the head oriented with the Frankfurt horizontal plane parallel to the floor.

The radiographs were performed using a Sirona Ortophos XG (Sirona Dental System, Inc., USA) with the following settings: a CCD sensor measuring 138 x 6.48 mm with 160 μm/pixel resolution, a tube voltage of 73 kV and a current of 15 mA, with 8.9 seconds of exposure time. The magnification factor reported by the manufacturer is 1.1.

Digital panoramic radiographs were excluded from the study when they showed: questionable styloid process, magnification errors and superimposition of other anatomical structure.

The styloid process length was measured using the measuring tool of Sidexis Software (Sirona Dental Systems, Inc., USA). It was measured from the point where it left the temporal bone plate to its tip (9). Styloid processes measuring more than 30 mm were considered elongated.

-Statistical analysis 

All data were entered into Excel 2010 (Microsoft, Remond, WA, USA). Statistical analyses were carried with the computing environment R version 3.2.1. that was used for estimating cross tabulation and the p-value of Chi-squared. The test was considered significant if the p-value was lower or equal to 0.05. Moreover it was used the R function fligner.test, that performs a Fligner-Killeen (median) test of the null that the variances in each of the groups (samples) are the same and the function shapiro.test that performs the Shapiro-Wilk test of normality. Then, we used the R oneway.test function that performs the t-test with Welch correction generalized to the case of arbitrarily many samples. Finally, the t-test was used to check a difference in mean between two samples if their variances were equal.

## Results

In the study sample 335 out 1003 subjects (33.40%) had radiographic images suggesting an elongated styloid process. 161 out 452 males (35.61%) and 174 out of 551 (31.57%) females patients showed an elongated styloid process. A *p*-value higher than 0.05 (0.177) showed no significant dependence of gender in styloid process elongation.

For what concerns the age groups ( Table 1), 15 out 178 patients (8.42%) under 17 years old, 62 out 186 (33.33%) patients between 18 and 35 years old, 79 out 214 (36.91%) and 179 out of 246 (72.76%) patients, respectively, between 36 and 53 and over 54 years old, that showed an elongated styloid process. We found a significant difference for what concerns the prevalence of the elongated styloid process among the age groups with a *p*-value lower than 0.05 (*p*-value<0.0001).

**Table 1:**
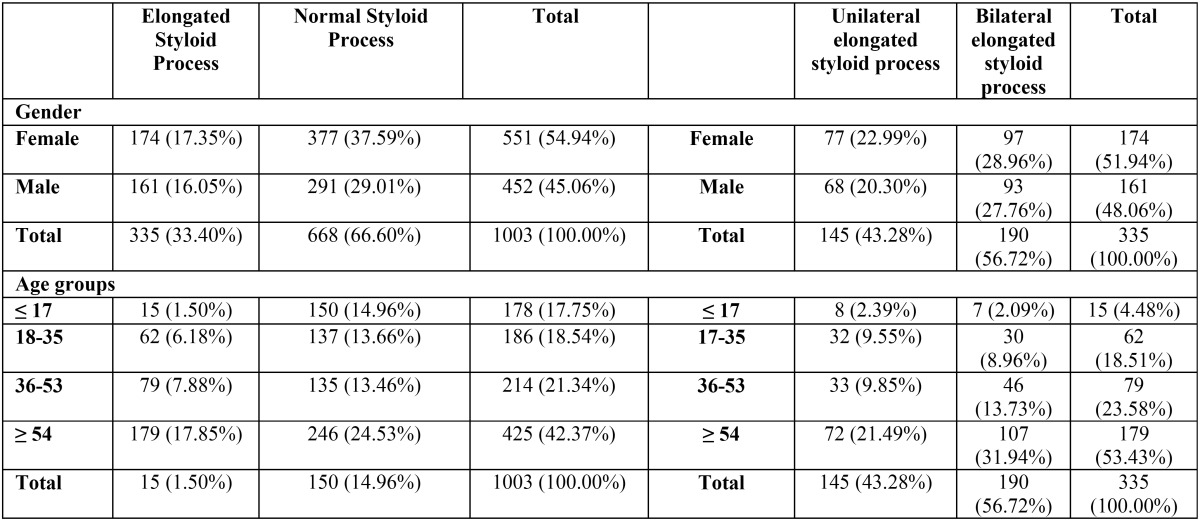
Absolute and relative frequencies of styloid processes according to gender and age.

Regarding differences bilateral and unilateral elongated styloid process ( Table 1), 190 subjects out of 335 (56.72 %) are elongated on both right and left side, while 145 styloid processes (43.28%) were elongated only in one side. No statistical significant was found between males and females, age groups and affected side with *p*-values higher than 0.05 (respectively 0.7097 and 0.5370).

 Table 2 shows the subjects split into the age groups and another variable that considers whether the left and right styloid processes are equal to zero or not. Note that the proportion of subjects having both styloid processes equal to 0 is different among age groups. Note that the 53.33% of the studied sample under 17 years old have both styloid processes equal to 0. A significance correlation between these two variables was found with a *p*-value lower than 0.05 (<0.0001).

**Table 2:**
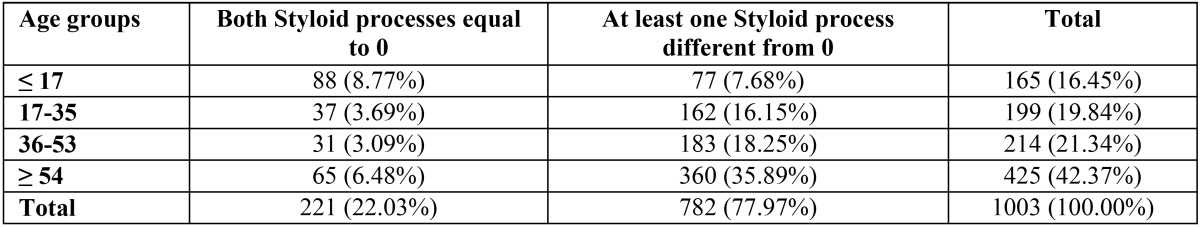
Absolute and relative frequencies of types of styloid processes according to age groups and styloid process elongation.

**Figure 1:**
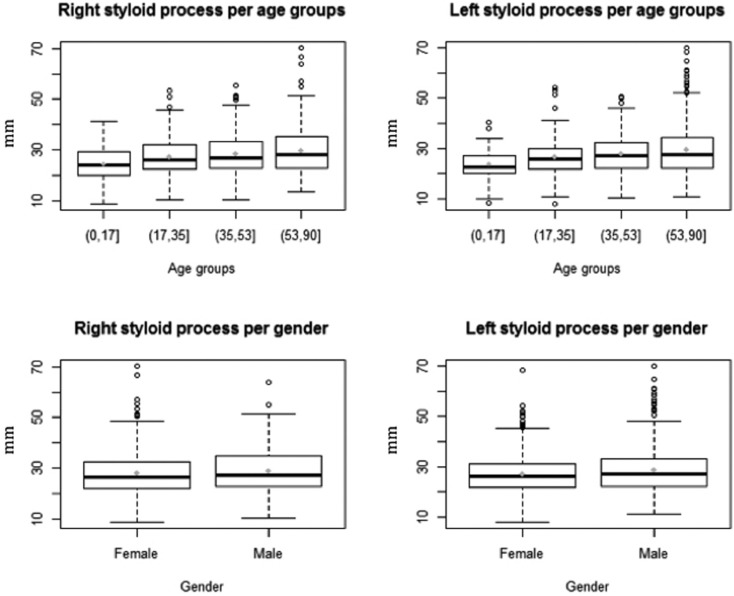
Right and left styloid process elongation distribution per age groups and gender represented by using boxplots. The means of each distribution are pointed out as a grey point.

The next analysis considers the right and left styloid process elongation as a continuous variable and tests and proposes an analy-sis of variance of these two continuous variables by age groups and gender excluding subjects presenting a styloid process elongation equal to 0 from the analysis ( Table 3). Figure 1 shows the right and left styloid process per age groups and gender while table III shows their means, medians and standard deviations per age groups, both of them do not consider subjects presenting a styloid process elongation equal to 0 from the analysis. The distributions are all slightly right skewed. Note also that the distributions do not present the same variance across the age groups. This can be tested by performing a Fligner-test that showed the variances in each age group are not the same for both right and left styloid process (respectively with p-values equal to 0.0210 and 0.0005). The same test was performed to detect the homoschedasticity of right and left styloid process in male and female groups. The tests did not reject the hypothesis of the equality of variances with *p*-values higher than 0.05 (respectively 0.62 and 0.07).

**Table 3:**
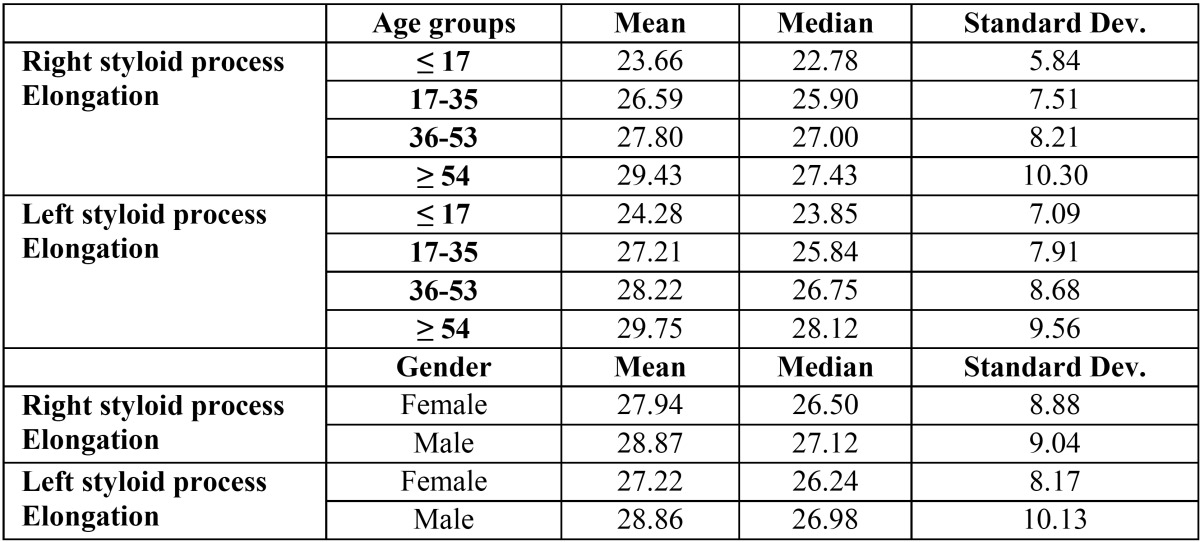
Mean, median and standard deviation per age groups and gender for the right and left styloid process elongation.

Moreover, the Shapiro test was performed in order to figure out whether the sample distributions of the right and left styloid process come from normal distributions. The age group that significantly comes from a normal distribution for both styloid processes is the one composed by subjects younger than 18 years old (*p*-value equal to 0.67 and 0.40 for right and left styloid elongated process, respectively). For the other groups the *p*-values were lower than 0.05, thus in favor of non-normality.

To test the equality of means of right and left styloid process among age groups, thus in a context of heterogeneity of variances and non-normal distributions, we applied the approximate method of Welch in the analysis of variance, which generalizes the commonly known 2-sample Welch test to the case of arbitrarily many samples. This method is particular indicated when distributions present heterogeneity of variance and with a slight right skewness.

Since the p-values are lower than 0.05 (*p*-value<0.0001) we can reject the null hypothesis that indicated that there is no difference in means of both styloid process among the age groups.

We calculated the pairwise comparisons between age groups by performing the Welch t-test. The p-values obtained were corrected by using the Holm correction for multiplicity and are showed in  Table 4 related to the right and left elongation process, respectively. Note that for both elongated process the mean of the age group composed by patients younger than 18 years old are significantly different compared to the means of the other age groups. Also, the right and left styloid process mean of the subject between 17 and 35 years old is significantly different than the mean of the patients older than 53 years old.

**Table 4:**
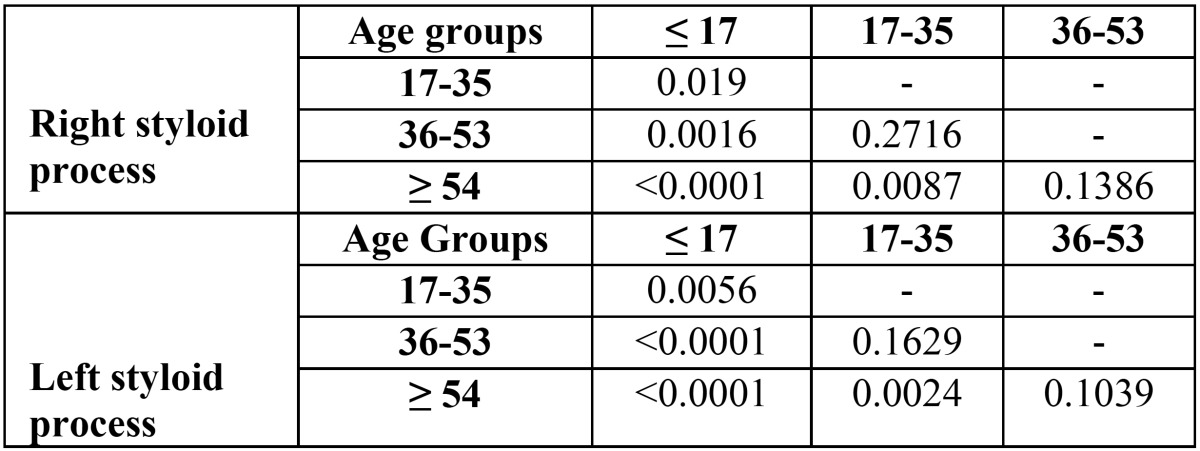
*p*-values of pairwise comparisons of right and left styloid process by using Welch t-test with Holm correction for multiplicity.

A t-test was performed in order the check whether the mean of the right and left styloid process are equal between male and fe-male subjects. In this case we did not use the Welch correction given that before we proved that the distributions have equal variances. We did not find a significant difference between male and female subjects in the right styloid process with a p-value hig-her than 0.05 (0.1616). On the contrary, for what concerns the left styloid process we found out a significance difference in mean between males and females with a p-value equal to 0.0177. Thus, the difference in mean between males and females subject for the left styloid process is not equal to 0 but is between 2.9142 and 0.2773 with a confidence interval of 95 percent.

## Discussion

The aim of this study was an epidemiological evaluation of the elongated styloid process on digital panoramic radiographs. This exam is easy to perform and to interpret and the lower radiant dose and cost than CT or CBCT suggest to use this exam for epidemiological evaluations.

The condition of elongated styloid process has been evaluated in various populations worldwide and its prevalence presents a high variability (4,10,11). In a previous study that evaluated the elongated styloid process in an Italian population a prevalence of 33% was found (5). Elongated styloid process can cause orofacial and neck pain during head rotational movement, deglutition and mouth opening and it characterizes the Eagle’syndrome (12).

Eagle’s syndrome shows different signs and symptoms like headache, otologia, dysphagia, throat pain, perception of a foreign body in the neck (13,14).

Eagle’s syndrome is often confused with some other orofacial disorders such as temporomandibular disorder, atypical orofacial pain, odontalgia and glossopharingeal neuralgia.

Some patients complain for a dental or temporomandibular treatment to solve their condition and dentists should diagnose the Eagle’s syndrome and refer the patients to the ENT.

The ENT could opt for a non-surgical treatment consisting in a pharmacological therapy with corticosteroids or topical infiltrations (15) even if most cases need a surgical approach for a complete resolution of the symptoms. Position, elongation and orientation are the parameters evaluated for an intraoral or extraoral approach.

In the present study 1003 patients between 5 and 90 years old were evaluated from the Complex Operating Unit of Dentistry of Padua University Hospital database and 33.40% of the patients present an elongated styloid process confirming the data previously reported in the medical literature showing a prevalence range between 1.4 to 83.6% (13,16,17) and the preliminary evaluation conducted at the University of Padua on a partial sample (5). The styloid process was measured from the point where it left the temporal bone plate to its tip, according with the method described by Ilguy *et al.* (9). No differences were found between males and females patients for every age group. This aspect show different results in medical literature: some studies reports a higher prevalence of elongated styloid process in males population (6,18), some others in females population (4,9) and some others report any difference between males and females (7,19). The lack of univocal consensus in medical literature suggests that gender is an irrelevant aspect in the etiology of the condition of the styloid process elongation.

In the different age group we found a progressive increase of the prevalence of the elongation with advancing age: 8.42% in the patients younger than 17 years old, 33.33% in the age group between 18 and 35 years old, 36.91% in the age group between 36 and 53 years old and 72.76% in the age group over 54 years old. Moreover, the right and left styloid process elongation was finally considered as a continuous variable and an increase in length of the process with the increasing age of the patients was found. Patients younger than 17 years old showed a mean length of 23.667 in the right side and 24.278 in the left side, the age group between 17 and 35 years old a mean length of 26.59 in the right side and 27.215 in the left side, the age group between 36 and 53 years old showed a mean length of 27.804 in the right side and 28.225 in the left side and patients older than 54 years old showed a mean length of 29.431 in the right side and 29.747 in the left side. Our results showed a slight difference between the left and the right side, but no statistically significant evidence was found. The increasing prevalence of the condition and the increasing mean length of the styloid process with advancing age confirm the chronic development of the calcification described in literature (12) even if a clear etiology has not already found.

In this study half of the patients present the elongation in the right side and the other half in the left side and for this reason there is no evidence of statistically significance difference on the side of the elongation.

When the styloid process is not evaluable in the radiograph, even if the area taken in the film is wide enough to include it, it means that the process is very short or anyway not calcified and the authors assigned it the value 0. The patients with both styloid process equal to 0 are differently distributed in the age groups: the 53.33% of the patients under 17 years old, the 18.59% between 17 and 35 years old, 14.49% between 36 and 53 years old, 15.29% in the patients older than 54 years old. Note that the decreasing of the patients with both styloid process equal to 0 with the increasing age is probably due to the increasing length of the process during the life of the patients; these results are in accordance with a study made by Rizzati-Barbosa *et al.* (12).

The authors suggest that further studies are needed using CT or CBCT for a three-dimension evaluation of the styloid process to investigate the relationship with other anatomical structures.

## Conclusions

This epidemiological study found an elongated styloid process in the 33.40% of the patients. The number of patients with elongated styloid process and the mean length of the process increase with the age confirming the chronic development of the calcification described in literature. No statistically significant correlation is found between the presence of elongated styloid process and the gender and affected side (bilateral or unilateral).
